# Factors influencing SMEs CloudERP adoption: A test with generalized linear model and artificial neural network

**DOI:** 10.1016/j.dib.2018.07.012

**Published:** 2018-07-11

**Authors:** Ahmad Habahbeh, Samson Oluwaseun Fadiya, Murat Akkaya

**Affiliations:** Department of Management Information Systems, Girne American University, Northern Cyprus, Cyprus

**Keywords:** CloudERP, Self-efficacy, Organizational support, Complexity, Compatibility, Perceived usefulness, Perceived ease of use, Performance expectancy, Facilitating conditions, Security, Relative advantage, Intention to use

## Abstract

This article present data concerning the factors influencing small and medium-sized enterprises (SMEs) intention to use/adopt CloudERP system in Jordan. Generalized Linear Modeling (GLM) and Artificial Neural Network (ANN) modeling techniques in R version 1.0.136 were used to analyze data obtained from 394 SMEs. Computer self-efficacy, organizational support, perceived usefulness, perceived ease of use, facilitating conditions, security and relative advantage have significant influence on the intention to use/adoption CloudERP systems. The survey data-set is made publicly available to amplify further inquiry.

**Specification Table**

**Table:** 

Subject area	Business, Management
*More Specific Subject Area*	*Enterprise Information systems*
*Type of Data*	*Text file, graph, figure, tables*
*How Data was Acquired*	*Survey*
*Data format*	*Raw*
*Experimental Factors*	*Sample consist of SMEs, authorities in these SMEs completed the survey.*
*Experimental features*	*Computer self-efficacy, organizational support, perceived usefulness, perceived ease of use, facilitating conditions, security and relative advantage have significant influence on the intention to use/adopt CloudERP systems*
*Data source location*	*Jordanian SMEs*
*Data Accessibility*	*Data is included in this article*

**Value of data**

• This data entails demographic characteristics of Jordanian SMEs.
• The data also describe the factors affecting the intention to use/adopt CloudERP systems among SMEs.
• Results from GLM and ANN suggest that computer self-efficacy, organizational support, perceived usefulness, perceived ease of use, facilitating conditions, security and relative advantage have significant influence on the intention to use/adopt CloudERP systems.

## 1 Data

Cloud computing is an emerging but distinct type of computing solution with the potentials to alter existing computing power, and deliver computing services differently in a new fashion and style. Cloud computing is an attractive bus stop and option for many SMEs due to its potentials, particularly in the present competitive world. Existing literature treated cloud computing and ERP as single and separate unit except [3], [5], [13]. Advances in technology has made modern organizations to integrate the systems as a single unit known as CloudERP. Research calls in CloudERP field were issued by [4], [5], [7], [9], [10]; this study is a compelling one, as it will address and fill the voids. This article examines the effects of computer self-efficacy, organizational support, relative advantage, security, performance expectancy, complexity, compatibility, perceived usefulness, and perceived ease of use on the intention to use/adoption CloudERP using generalized linear modeling and artificial neural network.

There are about 1400 registered SMEs in Amman, Jordan (http://www.jordanyp.com/category/Small_business/city:Amman). Survey monkey was used to evaluate the appropriate sample size (302 SMEs). A judgmental sampling approach subsumes issues like convenience and practicality and the errors of judgement in the selection will tend to counterbalance one another. We asked SMEs representative to participate in the study, these representatives include (owner, manager, and director). Overall, 394 representatives of SMEs participated in the study, the demographic breakdown is illustrated in Table 1.

**Table 1:** Demographic make-up.

	Frequency	Percentage
***Gender***		
Male	264	67.0
Female	130	33.0
Total	394	100.0
***Education***		
Some college degree	21	5.3
Bachelor׳s degree	327	83.0
Higher degree	46	11.7
Total	394	100.0
***Position***		
Owner	27	6.9
Manager	39	9.9
Director	328	83.2
Total	394	100.0
***Sector***		
Manufacturing	238	60.4
Wholesale and retail	156	39.6
Total	394	100.0
***Enterprise form of ownership***		
Sole proprietor	162	41.1
Partnership	152	38.6
Private company	80	20.3
Total	394	100.0

TL, Turkish Lira.

## 2 Experimental design, materials and methods

The data used in this study was collected via questionnaire and measures were adopted from prior researchers. Generalized Linear Modeling (GLM) and Artificial Neural Network (ANN) modeling techniques in R version 1.0.136 were used to analyze the data.

### 2.1 Computer self-efficacy

operationalized with ten items adopted from previous empirical work [6]. Sample item include: “I could complete the job using CloudERP if I had used similar packages before this one to do the same job”.

### 2.2 Organizational support

operationalized with three items adopted from previous empirical work [16]. Sample item include: “*In my company we get good technical support for our CloudERP system*”.

### 2.3 Complexity

operationalized with four items adopted from previous empirical work [16]. Sample item include: “*Using the CloudERP system involves much time doing mechanical operations (e.g., data input)*”.

### 2.4 Compatibility

operationalized with four items adopted from previous empirical work [16]. Sample item include: “*The changes caused by the adoption of CloudERP are compatible with the existing operating practices*”.

### 2.5 Perceived usefulness

operationalized with six items adopted from previous empirical work [8]. Sample item include: “CloudERP system would increase my productivity” and “CloudERP system would enhance my effectiveness”.

### 2.6 Perceive Ease of use

operationalized with six items adopted from previous empirical work [8]. Sample items include: “The CloudERP function is clear and understandable” and “CloudERP system is flexible to interact with”.

### 2.7 Performance expectancy

operationalized with three items adopted from previous empirical work [17]. Sample item include: “*Using the CloudERP system enables me to accomplish tasks more quickly*”.

### 2.8 Facilitating conditions

operationalized with three items adopted from previous empirical work [17]. Sample item include: “*I have the necessary resources to use CloudERP system”*.

### 2.9 Security (Information Integrity)

operationalized with two items adopted from previous empirical work [15]. Sample item include: “*Using CloudERP system would ensure the accuracy of the information handled*”.

### 2.10 Relative advantage

operationalized with four items adopted from previous empirical work [12]. Sample item include: “*CloudERP system would enable our enterprise to market our products/services in a better way”*.

### 2.11 Intention to use/adopt (IU)

operationalized with two items adopted from previous empirical work [14]. IU measures user׳s intent to use CloudERP system. Sample item include: “*I intend to use the CloudERP system for performing my job as often as needed*”.

The response choice for the variables was anchored on a 5-response choice Likert-type scale e.g., (1=strongly disagree) and (5=strongly agree). **Demographic** data includes gender, position in firm, sector of SMEs, level of education and structure of the SMEs

**Fig. 1:**
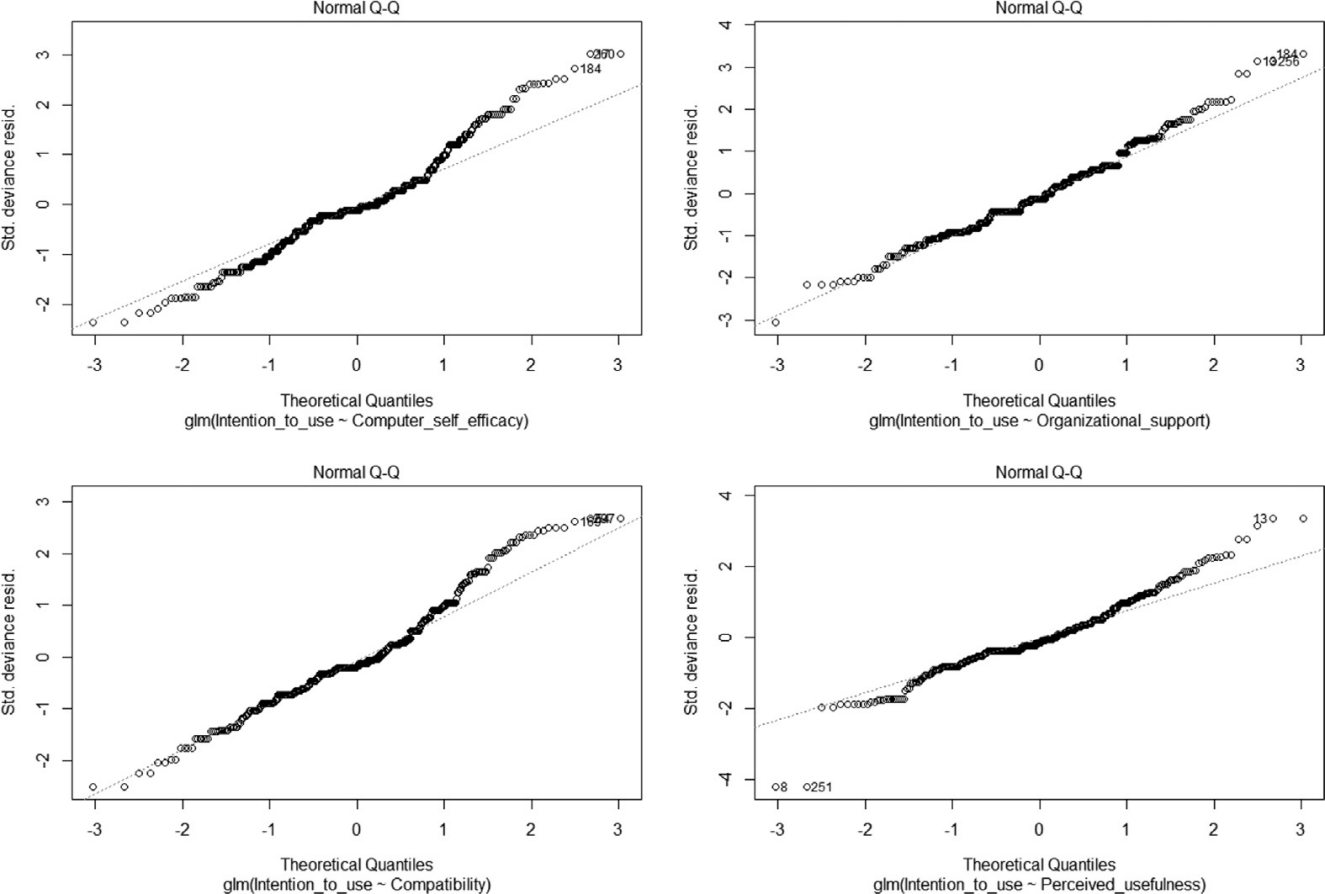
GLM graphs 1.

**Fig. 2:**
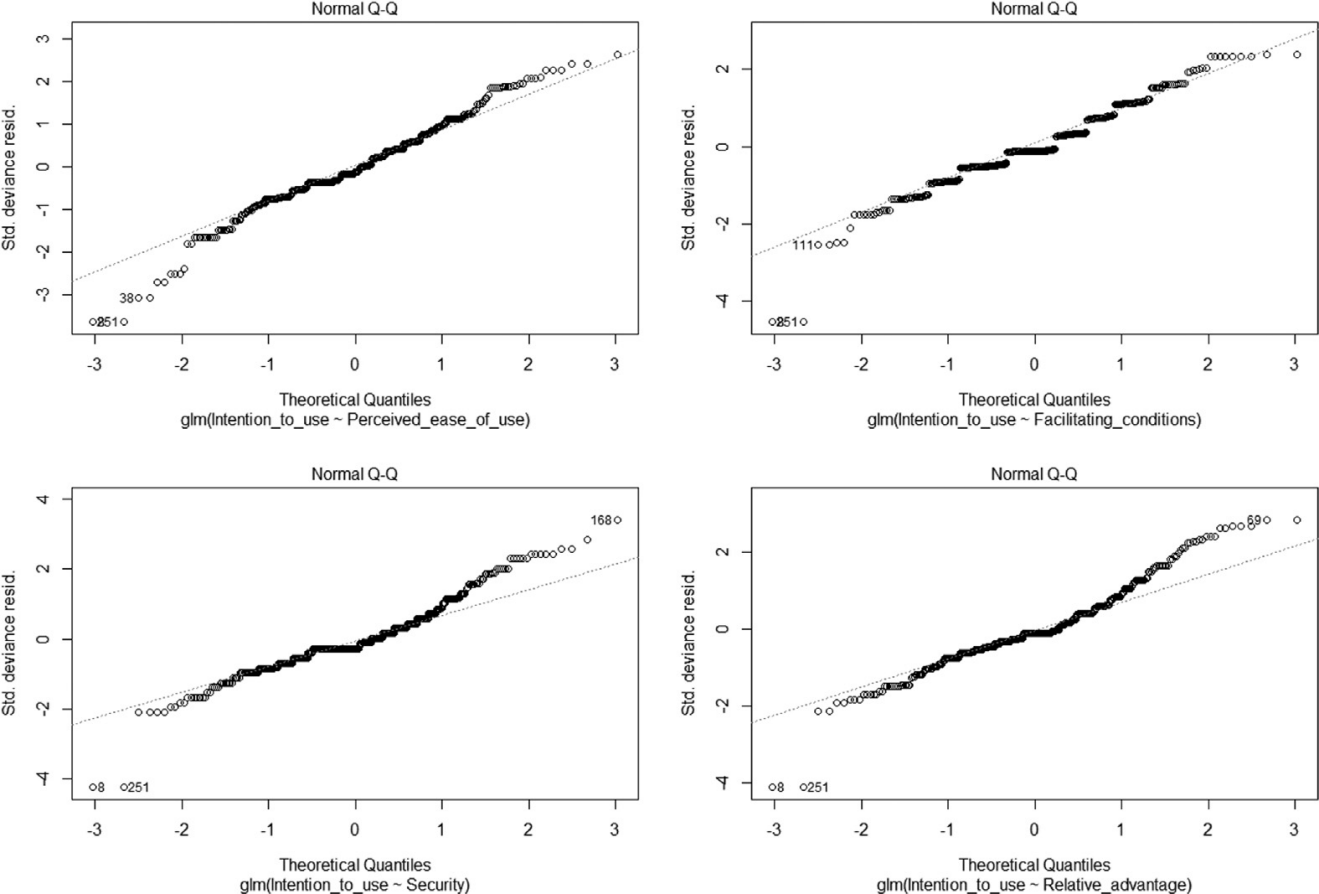
GLM graphs 2.

Generalized linear modeling (GLM) shows that all the predictors are significant, except complexity and performance expectancy that fail to exert significant effects. See Table 2, Fig. 1, Fig. 2. The R codes in Appendix was used for GLM and artificial neural network analysis.

**Table 2:** GLM Coefficients.

Variables	Estimate	Std. Error	t-value	Pr(>|t|)
(Intercept)	0.47788	0.15245	3.135	0.001853**
Computer self-efficacy	0.21302	0.05540	3.845	0.000141***
Organizational support	0.12409	0.04940	2.512	0.012410*
Complexity	−0.08023	0.05091	−1.576	0.115908
Compatibility	−0.18002	0.04925	−3.655	0.000293***
Perceived usefulness	−0.16302	0.06515	−2.502	0.012762*
Perceived ease of use	0.27793	0.06539	4.250	2.68e-05***
Performance expectancy	0.01394	0.05146	0.271	0.786627
Facilitating conditions	0.39176	0.05291	7.405	8.42e-13***
Security	0.11531	0.04334	2.661	0.008125**
Relative advantage	0.11713	0.05457	2.146	0.032474*
**Deviance residuals**:				
**Min**	**1Q**	**Median**	**3Q**	**Max**
−2.32745	−0.30272	−0.03011	0.27015	1.80280
Dispersion parameter for Gaussian family taken to be			: 0.2939605	
Null deviance			: 324.54 on 393 degrees of freedom	
Residual deviance			: 112.59 on 383 degrees of freedom	
AIC			: 648.59	
Number of Fisher Scoring iterations			: 2	
RMSE			: 0. 29	

Signif. Codes: 0 ‘***’ 0.001 ‘**’ 0.01 ‘*’ 0.05 ‘.’ 0.1 ‘’ 1.

Complexity and performance expectancy did not exert significant effects on the response variables in GLM modeling, as such these variables were excluded in ANN modeling. ANN outplays mainstream techniques such correlation analysis, linear and hierarchical regression, and even structural equation modeling. This is primarily due to its aptness to unmask linear and nonlinear association between variables, and its higher predictive accuracy in terms of relational effects. Withal, the issue of normality, linearity and homoscedasticity are not prerequisite in ANN as in traditional methods [1], [11]. Despite its remarkable power, the black-box nature of ANN limits its suitability in determining causal relationships. On the other hand, issues such as over-simplifying the complexities in decision making processes limits the suitability of linear techniques such as GLM and regression. Taken account of the above strengths and weakness, this study employed GLM and ANN to supplement each other and to augment this inquiry [2], [14]

**Fig. 3:**
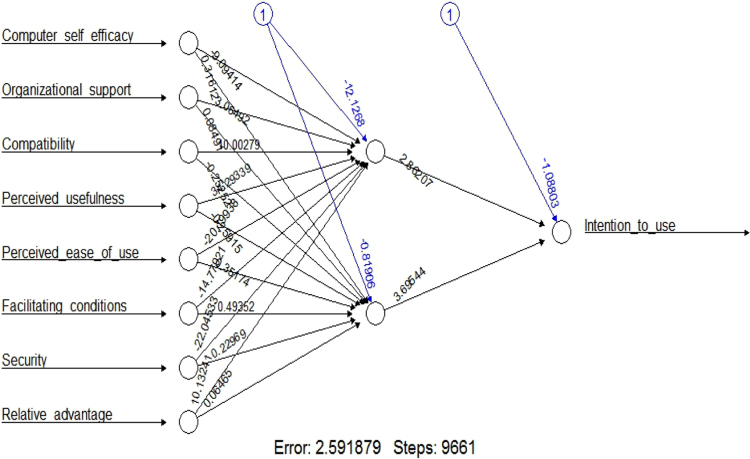
Artificial Neural network modeling.

Following prior scholars approach [1], [2], [11], “A Resilient Backpropagation with Weight Backtracking algorithm in R (neuralnet package) was used for the developed ANN model. Logistic function is used as the activation function for both hidden and output layer of the ANN model and sum squared errors (SSE) was used as differentiable error function with 2 hidden nodes”. The R codes used are provided in Appendix. Furthermore, GLM predicted a Mean Square of Error (MSE) that is equals to 0.29, while ANN prediction produced MSE that is equal to 0.01. This outcome suggest that ANN has a better prediction of the model. See Fig. 3.

**Fig. 4:**
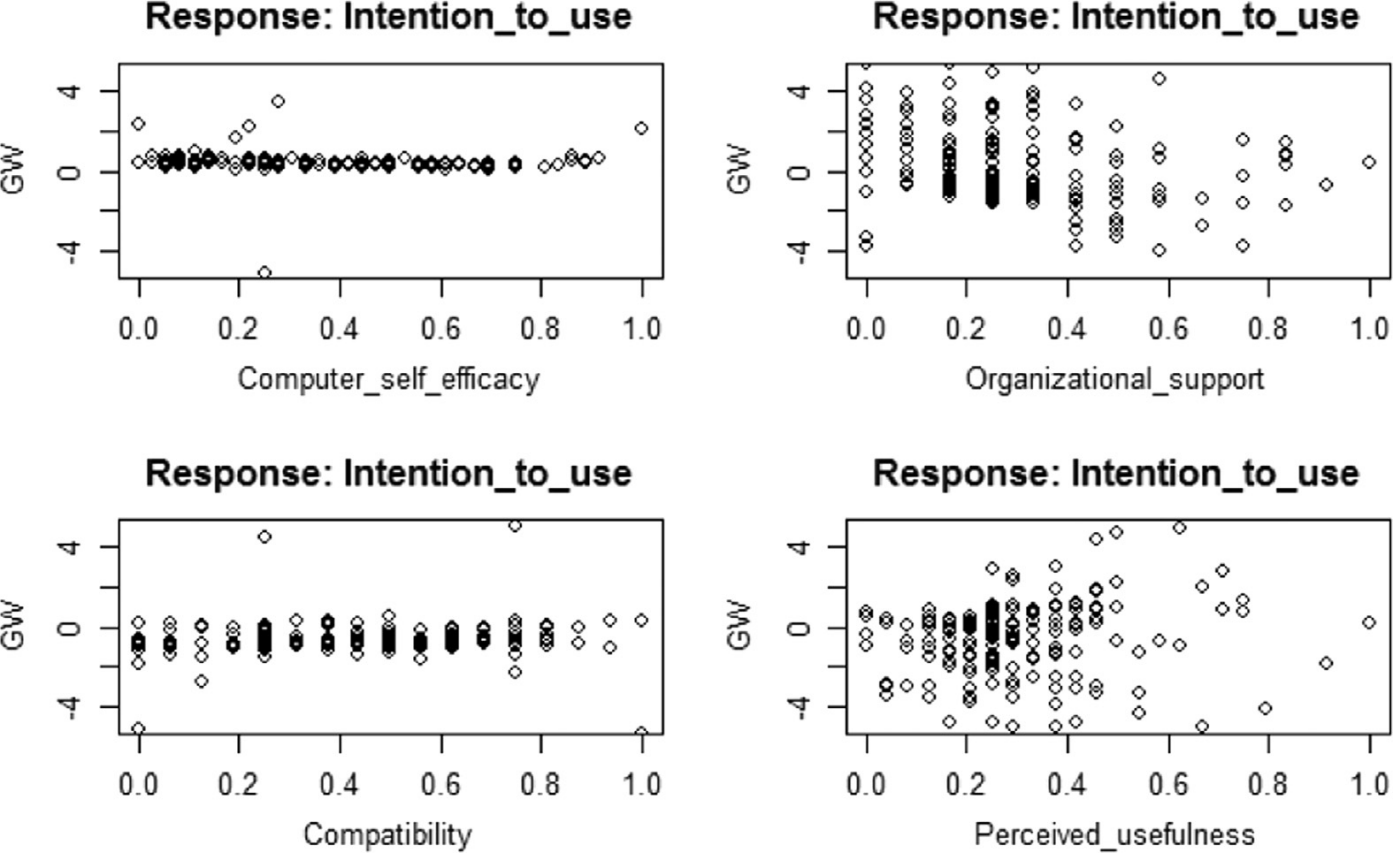
Artificial neural network modeling generalized weights diagram 1.

**Fig. 5:**
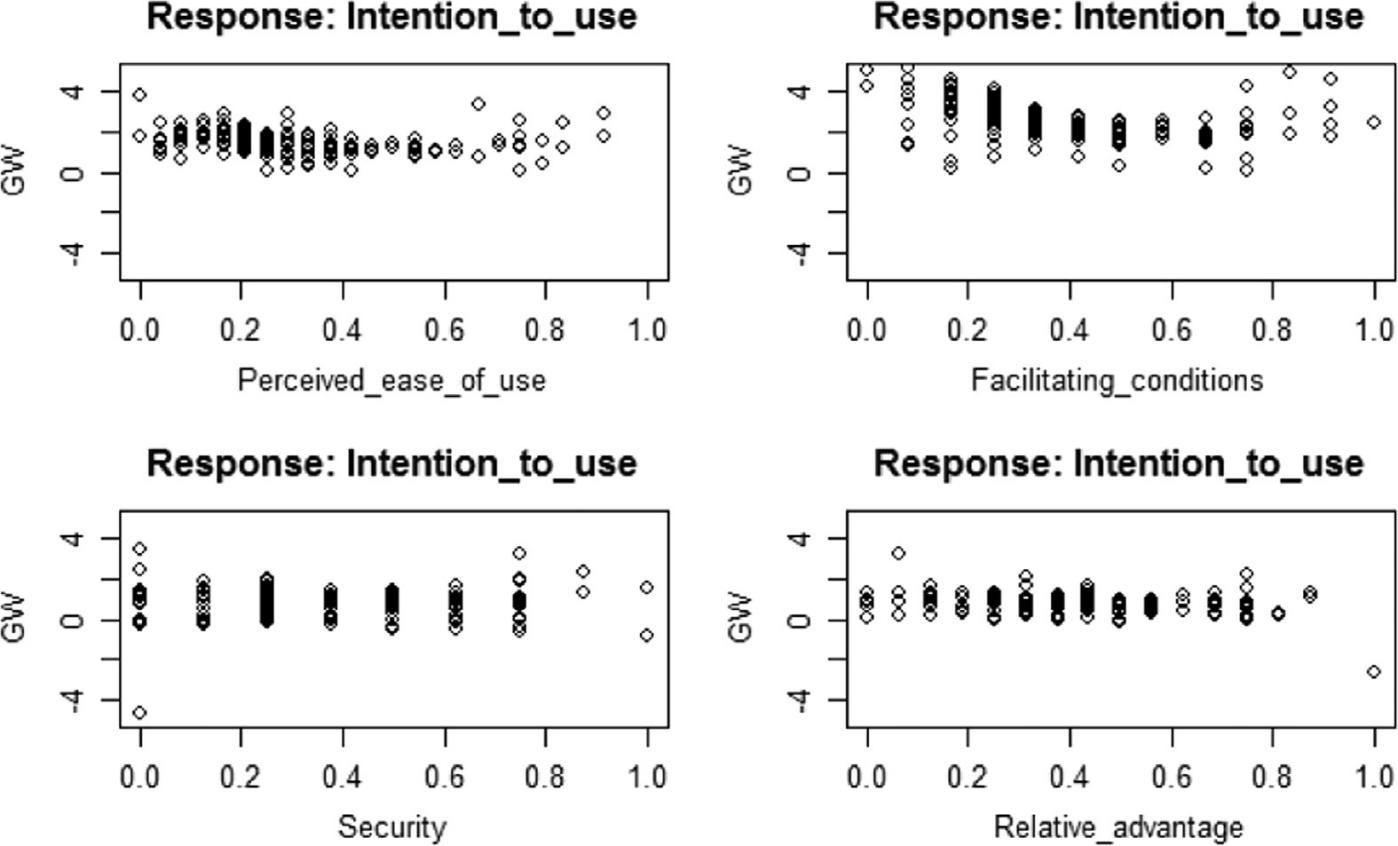
Artificial neural network modeling generalized weights diagram 2.

The training process required 9661 steps for all absolute partial derivatives of the error function to become less than 0.01. The distribution of the generalized weights delineates that computer self-efficacy, organizational support, perceived ease of use, facilitating conditions, security, and relative advantage have significant positive non-linear effects on SMEs intentions to use CloudERP solution. See Fig. 3, Fig. 4, Fig. 5. GLM shows that compatibility and perceived usefulness exerts negative linear effect on the intention to use /adopt CloudERP. In ANN this was true for compatibility as it exerts a negative non-linear effect, because majority of the weights were below 0.

However, perceived usefulness exerts both positive and negative non-linear effect, more specifically, low perceived usefulness has a negative effect on the intention to use/adopt CloudERP. Whereas, high perceived usefulness has a positive effect on the intention to use/adopt CloudERP. This provides confirmatory support for hypotheses [H1, H2, H5, H6, H8, H9 and H10]. Potential bias arising from over-fitting was evaded by cross-validation diagnoses in 10-folds, 75% of the data for training and 25% used for testing. The model accuracy was examined by comparing the MSE coefficients of the ten neural networks. Table 3 presents MSE coefficients of the ten neural networks, overall it seems that the model is predictive and reliable. This study has several limitations as follows: sample size is small, cross-sectional nature of the study, the outcome cannot be generalized to other countries with more advanced resources and regulations. The future of enterprise applications lies within the realm of CloudERP, as it presents several attractive and effective solutions to businesses.

**Table 3:** Neural network model performance.

Neural network #	Training	Testing
1	0.016	0.016
2	0.014	0.013
3	0.015	0.015
4	0.015	0.018
5	0.014	0.018
6	0.014	0.017
7	0.015	0.016
8	0.015	0.022
9	0.017	0.018
10	0.016	0.022
**Mean MSE**	0.015	0.017
