# Supplementary material for: Factors influencing SMEs CloudERP adoption: A test with generalized linear model and artificial neural network
Source: Data Brief. 2018 Jul 11;20:969–77. doi: 10.1016/j.dib.2018.07.012 (PMC6139002; doi:10.1016/j.dib.2018.07.012)
Supplement: Supplementary file 3 — Supplementary material [file mmc3.docx]

| # check for missing values  apply(Dataset,2,function(x) sum(is.na(x)))  # 75% for training and the rest for testing  index <- sample(1:nrow(Dataset),round(0.75*nrow(Dataset)))  train1 <- Dataset[index,]  test1 <- Dataset[-index,]  # appling GLM on the model  lm.fit <- glm (Intention_to_use ~ Computer_self_efficacy + Organizational_support + Complexity + Compatibility + Perceived_usefulness + Perceived_ease_of_use + Performance_expectancy + Facilitating_conditions + Security + Relative_advantage, data = Dataset)  # generating results (e.g. estimates and t-values)  summary(lm.fit)  #plotting functions  plot(lm.fit <- glm (Intention_to_use ~ Computer_self_efficacy, data = Dataset))  plot(lm.fit <- glm (Intention_to_use ~ Organizational_support, data = Dataset))  plot(lm.fit <- glm (Intention_to_use ~ Compatibility, data = Dataset))  plot(lm.fit <- glm (Intention_to_use ~ Perceived_usefulness, data = Dataset))  plot(lm.fit <- glm (Intention_to_use ~ Perceived_ease_of_use, data = Dataset))  plot(lm.fit <- glm (Intention_to_use ~ Facilitating_conditions, data = Dataset))  plot(lm.fit <- glm (Intention_to_use ~ Security, data = Dataset))  plot(lm.fit <- glm (Intention_to_use ~ Relative_advantage, data = Dataset))  # testing the model with the test dataset part  pr.lm <- predict(lm.fit,Dataset)  MSE.lm <- sum((pr.lm - Dataset$Intention_to_use)^2)/nrow(Dataset)  print(paste(MSE.lm))  #######################################  # NEURAL NET FITTİNG  #######################################  maxs <- apply(Dataset, 2, max)  mins <- apply(Dataset, 2, min)  scaled <- as.data.frame(scale(Dataset, center = mins, scale = maxs - mins))  train_ <- scaled[index,]  test_ <- scaled[-index,]  library(neuralnet)  nn <- neuralnet (Intention_to_use ~ Computer_self_efficacy + Organizational_support + Compatibility + Perceived_usefulness + Perceived_ease_of_use + Facilitating_conditions + Security + Relative_advantage, data = train_, hidden = 2, err.fct="sse",  linear.output=TRUE)  plot(nn)  nn$result.matrix  columns <- c("Computer_self_efficacy", "Organizational_support", "Compatibility", "Perceived_usefulness", "Perceived_ease_of_use", "Facilitating_conditions", "Security", "Relative_advantage")    covariate <- subset(test_ , select = columns)  pr.nn <- compute(nn, covariate, rep=1)  par(mfrow=c(2,2))  gwplot(nn, selected.covariate= "Computer_self_efficacy", selected.response = "Intention_to_use", min=-5, max=5)  gwplot(nn, selected.covariate= "Organizational_support", selected.response = "Intention_to_use", min=-5, max=5)  gwplot(nn, selected.covariate= "Compatibility", selected.response = "Intention_to_use", min=-5, max=5)  gwplot(nn, selected.covariate= "Perceived_usefulness", selected.response = "Intention_to_use", min=-5, max=5)  gwplot(nn, selected.covariate= "Perceived_ease_of_use", selected.response = "Intention_to_use", min=-5, max=5)  gwplot(nn, selected.covariate= "Facilitating_conditions", selected.response = "Intention_to_use", min=-5, max=5)  gwplot(nn, selected.covariate= "Security", selected.response = "Intention_to_use", min=-5, max=5)  gwplot(nn, selected.covariate= "Relative_advantage", selected.response = "Intention_to_use", min=-5, max=5)  # Next step  pr.nn_ <- pr.nn$net.result*(max(test_$Intention_to_use)-min(test_$Intention_to_use))+min(test_$Intention_to_use)  test.r <- (test_$Intention_to_use)*(max(test_$Intention_to_use)-min(test_$Intention_to_use))+min(test_$Intention_to_use)    # Calculating MSE  MSE.nn <- sum((test.r - pr.nn_)^2)/nrow(test_)  #Compare the two MSEs  print(paste(MSE.lm, MSE.nn))    ############################FOR TRAİNİNG###########################  set.seed(450)  cv.error <- NULL  k <- 10  library(plyr)  pbar <- create_progress_bar('text')  pbar$init(k)  for(i in 1:k)  {  index <- sample(1:nrow(Dataset),round(0.75*nrow(Dataset)))  train.cv <- scaled[index,]  test.cv <- scaled[-index,]    library(neuralnet)  nn <- neuralnet (Intention_to_use ~ Computer_self_efficacy + Organizational_support + Compatibility + Perceived_usefulness + Perceived_ease_of_use +Facilitating_conditions + Security + Relative_advantage, data = train.cv, hidden = 2, err.fct="sse", linear.output=TRUE)  columns <- c("Computer_self_efficacy", "Organizational_support", "Compatibility", "Perceived_usefulness", "Perceived_ease_of_use", "Facilitating_conditions", "Security", "Relative_advantage")    covariate <- subset(train.cv , select = columns)  pr.nn <- compute(nn, covariate, rep=1)  pr.nn <- pr.nn$net.result*(max(train.cv$Intention_to_use)-min(train.cv$Intention_to_use))+min(train.cv$Intention_to_use)    train.cv.r <- (train.cv$Intention_to_use)*(max(train.cv$Intention_to_use)min(train.cv$Intention_to_use))+min(train.cv$Intention_to_use)  cv.error[i] <- sum((train.cv.r - pr.nn)^2)/nrow(train.cv)  print(paste(cv.error[i]))  pbar$step()  }  mean(cv.error)  ############################FOR TESTİNG###########################  set.seed(450)  cv.error <- NULL  k <- 10  library(plyr)  pbar <- create_progress_bar('text')  pbar$init(k)  for(i in 1:k)  {  index <- sample(1:nrow(Dataset),round(0.75*nrow(Dataset)))  train.cv <- scaled[index,]  test.cv <- scaled[-index,]    library(neuralnet)  nn <- neuralnet (Intention_to_use ~ Computer_self_efficacy + Organizational_support + Compatibility + Perceived_usefulness + Perceived_ease_of_use +Facilitating_conditions + Security + Relative_advantage,  data = train.cv, hidden = 2, err.fct="sse", linear.output=TRUE)  columns <- c("Computer_self_efficacy", "Organizational_support", "Compatibility", "Perceived_usefulness",  "Perceived_ease_of_use", "Facilitating_conditions", "Security", "Relative_advantage")    covariate <- subset(test.cv , select = columns)  pr.nn <- compute(nn, covariate, rep=1)  pr.nn <- pr.nn$net.result*(max(test.cv$Intention_to_use)-min(test.cv$Intention_to_use))+min(test.cv$Intention_to_use)    test.cv.r <- (test.cv$Intention_to_use)*(max(test.cv$Intention_to_use)-min(test.cv$Intention_to_use))+min(test.cv$Intention_to_use)  cv.error[i] <- sum((test.cv.r - pr.nn)^2)/nrow(test.cv)  print(paste(cv.error[i]))    pbar$step()  }  mean(cv.error) |
| --- |
