# Supplementary material for: Factors influencing SMEs CloudERP adoption: A test with generalized linear model and artificial neural network
Source: Data Brief. 2018 Jul 11;20:969–77. doi: 10.1016/j.dib.2018.07.012 (PMC6139002; doi:10.1016/j.dib.2018.07.012)
Supplement: Supplementary file 4 — Supplementary material [file mmc4.pdf]

Package ‘neuralnet’

August 16, 2016

Type Package

Title Training of Neural Networks

Version 1.33

Date 2016-08-05

Author Stefan Fritsch [aut], Frauke Guenther [aut, cre], Marc Suling [ctb], Sebastian M. Mueller [ctb]

Maintainer Frauke Guenther <guenther@leibniz-bips.de>

Depends R (>= 2.9.0)

Imports grid, MASS, grDevices, stats, utils

Description Training of neural networks using backpropagation, resilient backpropagation with (Riedmiller, 1994) or without weight backtracking (Riedmiller and Braun, 1993) or the modified globally convergent version by Anastasiadis et al. (2005). The package allows flexible settings through custom-choice of error and activation function. Furthermore, the calculation of generalized weights (Intrator O & Intrator N, 1993) is implemented.

License GPL (>= 2)

NeedsCompilation no

Repository CRAN

Date/Publication 2016-08-16 12:08:44

R topics documented:

neralnet-package 2
compute 3
confidence.interval 4
gwplot 6
neuralnet 7
plot.nn 10
prediction 12

Index **13**

neuralnet-package *Training of Neural Networks*

Description

Training of neural networks using the backpropagation, resilient backpropagation with (Riedmiller, 1994) or without weight backtracking (Riedmiller, 1993) or the modified globally convergent version by Anastasiadis et al. (2005). The package allows flexible settings through custom-choice of error and activation function. Furthermore, the calculation of generalized weights (Intrator O & Intrator N, 1993) is implemented.

Details

Package:	neuralnet
Type:	Package
Version:	1.33
Date:	2016-08-05
License:	GPL (>=2)

Note

This work has been supported by the German Research Foundation
(DFG: http://www.dfg.de) under grant scheme PI 345/3-1.

Author(s)

Stefan Fritsch, Frauke Guenther <guenther@leibniz-bips.de>,

Maintainer: Frauke Guenther <guenther@leibniz-bips.de>

References

Riedmiller M. (1994) *Rprop - Description and Implementation Details.* Technical Report. University of Karlsruhe.

Riedmiller M. and Braun H. (1993) *A direct adaptive method for faster backpropagation learning: The RPROP algorithm.* Proceedings of the IEEE International Conference on Neural Networks (ICNN), pages 586-591. San Francisco.

Anastasiadis A. et. al. (2005) *New globally convergent training scheme based on the resilient propagation algorithm.* Neurocomputing 64, pages 253-270.

Intrator O. and Intrator N. (1993) *Using Neural Nets for Interpretation of Nonlinear Models.* Proceedings of the Statistical Computing Section, 244-249 San Francisco: American Statistical Society (eds).

See Also

plot.nn for plotting of the neural network.

gwplot for plotting of the generalized weights.

compute for computation of the calculated network.

confidence.interval for calculation of a confidence interval for the weights.

prediction for calculation of a prediction.

Examples

```
AND <- c(rep(0,7),1)
OR <- c(0,rep(1,7))
binary.data <- data.frame(expand.grid(c(0,1), c(0,1), c(0,1)), AND, OR)
print(net <- neuralnet(AND+OR~Var1+Var2+Var3,  binary.data, hidden=0,
             rep=10, err.fct="ce", linear.output=FALSE))

XOR <- c(0,1,1,0)
xor.data <- data.frame(expand.grid(c(0,1), c(0,1)), XOR)
print(net.xor <- neuralnet(XOR~Var1+Var2, xor.data, hidden=2, rep=5))
plot(net.xor, rep="best")

data(infert, package="datasets")
print(net.infert <- neuralnet(case~parity+induced+spontaneous,  infert,
                    err.fct="ce", linear.output=FALSE, likelihood=TRUE))
gwplot(net.infert, selected.covariate="parity")
gwplot(net.infert, selected.covariate="induced")
gwplot(net.infert, selected.covariate="spontaneous")
confidence.interval(net.infert)

Var1 <- runif(50, 0, 100)
sqrt.data <- data.frame(Var1, Sqrt=sqrt(Var1))
print(net.sqrt <- neuralnet(Sqrt~Var1, sqrt.data, hidden=10,
                  threshold=0.01))
compute(net.sqrt, (1:10)^2)$net.result

Var1 <- rpois(100,0.5)
Var2 <- rbinom(100,2,0.6)
Var3 <- rbinom(100,1,0.5)
SUM <- as.integer(abs(Var1+Var2+Var3+(rnorm(100))))
sum.data <- data.frame(Var1+Var2+Var3, SUM)
print(net.sum <- neuralnet(SUM~Var1+Var2+Var3, sum.data, hidden=1,
                 act.fct="tanh"))
prediction(net.sum)
```

---

compute *Computation of a given neural network for given covariate vectors*

Description

`compute`, a method for objects of class `nn`, typically produced by `neuralnet`. Computes the outputs of all neurons for specific arbitrary covariate vectors given a trained neural network. Please make sure that the order of the covariates is the same in the new matrix or dataframe as in the original neural network.

Usage

```
compute(x, covariate, rep = 1)
```

Arguments

`x`	an object of class `nn`.
`covariate`	a dataframe or matrix containing the variables that had been used to train the neural network.
`rep`	an integer indicating the neural network's repetition which should be used.

Value

`compute` returns a list containing the following components:

`neurons`	a list of the neurons' output for each layer of the neural network.
`net.result`	a matrix containing the overall result of the neural network.

Author(s)

Stefan Fritsch, Frauke Guenther <guenther@leibniz-bips.de>

Examples

```
Var1 <- runif(50, 0, 100)
sqrt.data <- data.frame(Var1, Sqrt=sqrt(Var1))
print(net.sqrt <- neuralnet(Sqrt~Var1,  sqrt.data, hidden=10,
                  threshold=0.01))
compute(net.sqrt, (1:10)^2)$net.result
```

`confidence.interval` *Calculates confidence intervals of the weights*

Description

`confidence.interval`, a method for objects of class `nn`, typically produced by `neuralnet`. Calculates confidence intervals of the weights (White, 1989) and the network information criteria NIC (Murata et al. 1994). All confidence intervals are calculated under the assumption of a local identification of the given neural network. If this assumption is violated, the results will not be reasonable. Please make also sure that the chosen error function equals the negative log-likelihood function, otherwise the results are not meaningfull, too.

Usage

```
confidence.interval(x, alpha = 0.05)
```

Arguments

`x`	neural network
`alpha`	numerical. Sets the confidence level to (1-alpha).

Value

`confidence.interval` returns a list containing the following components:

`lower.ci`	a list containing the lower confidence bounds of all weights of the neural network differentiated by the repetitions.
`upper.ci`	a list containing the upper confidence bounds of all weights of the neural network differentiated by the repetitions.
`nic`	a vector containg the information criteria NIC for every repetition.

Author(s)

Stefan Fritsch, Frauke Guenther <guenther@leibniz-bips.de>

References

White (1989) *Learning in artificial neural networks. A statistical perspective.* Neural Computation (1), pages 425-464

Murata et al. (1994) *Network information criterion - determining the number of hidden units for an artificial neural network model.* IEEE Transactions on Neural Networks 5 (6), pages 865-871

See Also

`neuralnet`

Examples

```
data(infert, package="datasets")
print(net.infert <- neuralnet(case~parity+induced+spontaneous,
                    infert, err.fct="ce", linear.output=FALSE))
confidence.interval(net.infert)
```

gwplot *Plot method for generalized weights*

Description

gwplot, a method for objects of class nn, typically produced by neuralnet. Plots the generalized weights (Intrator and Intrator, 1993) for one specific covariate and one response variable.

Usage

```
gwplot(x, rep = NULL, max = NULL, min = NULL, file = NULL,
       selected.covariate = 1, selected.response = 1,
       highlight = FALSE, type="p", col = "black", ...)
```

Arguments

x	an object of class nn
rep	an integer indicating the repetition to plot. If rep="best", the repetition with the smallest error will be plotted. If not stated all repetitions will be plotted.
max	maximum of the y axis. In default, max is set to the highest y-value.
min	minimum of the y axis. In default, min is set to the smallest y-value.
file	a character string naming the plot to write to. If not stated, the plot will not be saved.
selected.covariate	either a string of the covariate's name or an integer of the ordered covariates, indicating the reference covariate in the generalized weights plot. Defaulting to the first covariate.
selected.response	either a string of the response variable's name or an integer of the ordered response variables, indicating the reference response in the generalized weights plot. Defaulting to the first response variable.
highlight	a logical value, indicating whether to highlight (red color) the best repetition (smallest error). Only reasonable if rep=NULL. Default is FALSE
type	a character indicating the type of plotting; actually any of the types as in plot.default.
col	a color of the generalized weights.
...	Arguments to be passed to methods, such as graphical parameters (see par).

Author(s)

Stefan Fritsch, Frauke Guenther <guenther@leibniz-bips.de>

References

Intrator O. and Intrator N. (1993) *Using Neural Nets for Interpretation of Nonlinear Models.* Proceedings of the Statistical Computing Section, 244-249 San Francisco: American Statistical Society (eds.)

See Also

neuralnet

Examples

```
data(infert, package="datasets")
print(net.infert <- neuralnet(case~parity+induced+spontaneous, infert,
                err.fct="ce", linear.output=FALSE, likelihood=TRUE))
gwplot(net.infert, selected.covariate="parity")
gwplot(net.infert, selected.covariate="induced")
gwplot(net.infert, selected.covariate="spontaneous")
```

---

neuralnet *Training of neural networks*

Description

neuralnet is used to train neural networks using backpropagation, resilient backpropagation (RPROP) with (Riedmiller, 1994) or without weight backtracking (Riedmiller and Braun, 1993) or the modified globally convergent version (GRPROP) by Anastasiadis et al. (2005). The function allows flexible settings through custom-choice of error and activation function. Furthermore the calculation of generalized weights (Intrator O. and Intrator N., 1993) is implemented.

Usage

```
neuralnet(formula, data, hidden = 1, threshold = 0.01,
          stepmax = 1e+05, rep = 1, startweights = NULL,
          learningrate.limit = NULL,
          learningrate.factor = list(minus = 0.5, plus = 1.2),
          learningrate=NULL, lifesign = "none",
          lifesign.step = 1000, algorithm = "rprop+",
          err.fct = "sse", act.fct = "logistic",
          linear.output = TRUE, exclude = NULL,
          constant.weights = NULL, likelihood = FALSE)
```

Arguments

formula	a symbolic description of the model to be fitted.
data	a data frame containing the variables specified in formula.
hidden	a vector of integers specifying the number of hidden neurons (vertices) in each layer.
threshold	a numeric value specifying the threshold for the partial derivatives of the error function as stopping criteria.
stepmax	the maximum steps for the training of the neural network. Reaching this maximum leads to a stop of the neural network's training process.
rep	the number of repetitions for the neural network's training.

`startweights` a vector containing starting values for the weights. The weights will not be randomly initialized.

`learningrate.limit`
a vector or a list containing the lowest and highest limit for the learning rate. Used only for RPROP and GRPROP.

`learningrate.factor`
a vector or a list containing the multiplication factors for the upper and lower learning rate. Used only for RPROP and GRPROP.

`learningrate` a numeric value specifying the learning rate used by traditional backpropagation. Used only for traditional backpropagation.

`lifesign` a string specifying how much the function will print during the calculation of the neural network. 'none', 'minimal' or 'full'.

`lifesign.step` an integer specifying the stepsize to print the minimal threshold in full lifesign mode.

`algorithm` a string containing the algorithm type to calculate the neural network. The following types are possible: 'backprop', 'rprop+', 'rprop-', 'sag', or 'slr'. 'backprop' refers to backpropagation, 'rprop+' and 'rprop-' refer to the resilient backpropagation with and without weight backtracking, while 'sag' and 'slr' induce the usage of the modified globally convergent algorithm (grprop). See Details for more information.

`err.fct` a differentiable function that is used for the calculation of the error. Alternatively, the strings 'sse' and 'ce' which stand for the sum of squared errors and the cross-entropy can be used.

`act.fct` a differentiable function that is used for smoothing the result of the cross product of the covariate or neurons and the weights. Additionally the strings, 'logistic' and 'tanh' are possible for the logistic function and tangent hyperbolicus.

`linear.output` logical. If act.fct should not be applied to the output neurons set linear output to TRUE, otherwise to FALSE.

`exclude` a vector or a matrix specifying the weights, that are excluded from the calculation. If given as a vector, the exact positions of the weights must be known. A matrix with n-rows and 3 columns will exclude n weights, where the first column stands for the layer, the second column for the input neuron and the third column for the output neuron of the weight.

`constant.weights`
a vector specifying the values of the weights that are excluded from the training process and treated as fix.

`likelihood` logical. If the error function is equal to the negative log-likelihood function, the information criteria AIC and BIC will be calculated. Furthermore the usage of confidence.interval is meaningfull.

Details

The globally convergent algorithm is based on the resilient backpropagation without weight backtracking and additionally modifies one learning rate, either the learningrate associated with the smallest absolute gradient (sag) or the smallest learningrate (slr) itself. The learning rates in the grprop algorithm are limited to the boundaries defined in learningrate.limit.

Value

`neuralnet` returns an object of class `nn`. An object of class `nn` is a list containing at most the following components:

`call` the matched call.

`response` extracted from the `data argument`.

`covariate` the variables extracted from the `data argument`.

`model.list` a list containing the covariates and the response variables extracted from the `formula argument`.

`err.fct` the error function.

`act.fct` the activation function.

`data` the `data argument`.

`net.result` a list containing the overall result of the neural network for every repetition.

`weights` a list containing the fitted weights of the neural network for every repetition.

`generalized.weights`
a list containing the generalized weights of the neural network for every repetition.

`result.matrix`
a matrix containing the reached threshold, needed steps, error, AIC and BIC (if computed) and weights for every repetition. Each column represents one repetition.

`startweights` a list containing the startweights of the neural network for every repetition.

Author(s)

Stefan Fritsch, Frauke Guenther `<guenther@leibniz-bips.de>`

References

Riedmiller M. (1994) *Rprop - Description and Implementation Details.* Technical Report. University of Karlsruhe.

Riedmiller M. and Braun H. (1993) *A direct adaptive method for faster backpropagation learning: The RPROP algorithm.* Proceedings of the IEEE International Conference on Neural Networks (ICNN), pages 586-591. San Francisco.

Anastasiadis A. et. al. (2005) *New globally convergent training scheme based on the resilient propagation algorithm.* Neurocomputing 64, pages 253-270.

Intrator O. and Intrator N. (1993) *Using Neural Nets for Interpretation of Nonlinear Models.* Proceedings of the Statistical Computing Section, 244-249 San Francisco: American Statistical Society (eds).

See Also

plot.nn for plotting the neural network.

gwplot for plotting the generalized weights.

compute for computation of a given neural network for given covariate vectors.

confidence.interval for calculation of confidence intervals of the weights.

prediction for a summary of the output of the neural network.

Examples

```
AND <- c(rep(0,7),1)
OR <- c(0,rep(1,7))
binary.data <- data.frame(expand.grid(c(0,1), c(0,1), c(0,1)), AND, OR)
print(net <- neuralnet(AND+OR~Var1+Var2+Var3,  binary.data, hidden=0,
             rep=10, err.fct="ce", linear.output=FALSE))

data(infert, package="datasets")
print(net.infert <- neuralnet(case~parity+induced+spontaneous, infert,
                    err.fct="ce", linear.output=FALSE, likelihood=TRUE))
```

---

plot.nn *Plot method for neural networks*

Description

plot.nn, a method for the plot generic. It is designed for an inspection of the weights for objects of class nn, typically produced by neuralnet.

Usage

```
    ## S3 method for class 'nn'
plot(x, rep = NULL, x.entry = NULL, x.out = NULL,
        radius = 0.15, arrow.length = 0.2, intercept = TRUE,
        intercept.factor = 0.4, information = TRUE,
        information.pos = 0.1, col.entry.synapse = "black",
        col.entry = "black", col.hidden = "black",
        col.hidden.synapse = "black", col.out = "black",
        col.out.synapse = "black", col.intercept = "blue",
        fontsize = 12, dimension = 6, show.weights = TRUE,
        file = NULL, ...)
```

Arguments

x	an object of class nn
rep	repetition of the neural network. If rep="best", the repetition with the smallest error will be plotted. If not stated all repetitions will be plotted, each in a separate window.

`x.entry`	x-coordinate of the entry layer. Depends on the arrow.length in default.
`x.out`	x-coordinate of the output layer.
`radius`	radius of the neurons.
`arrow.length`	length of the entry and out arrows.
`intercept`	a logical value indicating whether to plot the intercept.
`intercept.factor`	x-position factor of the intercept. The closer the factor is to 0, the closer the intercept is to its left neuron.
`information`	a logical value indicating whether to add the error and steps to the plot.
`information.pos`	y-position of the information.
`col.entry.synapse`	color of the synapses leading to the input neurons.
`col.entry`	color of the input neurons.
`col.hidden`	color of the neurons in the hidden layer.
`col.hidden.synapse`	color of the weighted synapses.
`col.out`	color of the output neurons.
`col.out.synapse`	color of the synapses leading away from the output neurons.
`col.intercept`	color of the intercept.
`fontsize`	fontsize of the text.
`dimension`	size of the plot in inches.
`show.weights`	a logical value indicating whether to print the calculated weights above the synapses.
`file`	a character string naming the plot to write to. If not stated, the plot will not be saved.
`...`	arguments to be passed to methods, such as graphical parameters (see `par`).

Author(s)

Stefan Fritsch, Frauke Guenther <guenther@leibniz-bips.de>

See Also

`neuralnet`

Examples

```
XOR <- c(0,1,1,0)
xor.data <- data.frame(expand.grid(c(0,1), c(0,1)), XOR)
print(net.xor <- neuralnet( XOR~Var1+Var2, xor.data, hidden=2, rep=5))
plot(net.xor, rep="best")
```

`prediction` *Summarizes the output of the neural network, the data and the fitted values of glm objects (if available)*

Description

`prediction`, a method for objects of class `nn`, typically produced by `neuralnet`. In a first step, the dataframe will be amended by a mean response, the mean of all responses corresponding to the same covariate-vector. The calculated data.error is the error function between the original response and the new mean response. In a second step, all duplicate rows will be erased to get a quick overview of the data. To obtain an overview of the results of the neural network and the glm objects, the covariate matrix will be bound to the output of the neural network and the fitted values of the glm object(if available) and will be reduced by all duplicate rows.

Usage

```
prediction(x, list.glm = NULL)
```

Arguments

`x`	neural network
`list.glm`	an optional list of glm objects

Value

a list of the summaries of the repetitions of the neural networks, the data and the glm objects (if available).

Author(s)

Stefan Fritsch, Frauke Guenther <guenther@leibniz-bips.de>

See Also

`neuralnet`

Examples

```
Var1 <- rpois(100,0.5)
Var2 <- rbinom(100,2,0.6)
Var3 <- rbinom(100,1,0.5)
SUM <- as.integer(abs(Var1+Var2+Var3+(rnorm(100))))
sum.data <- data.frame(Var1+Var2+Var3, SUM)
print(net.sum <- neuralnet( SUM~Var1+Var2+Var3,  sum.data, hidden=1,
                 act.fct="tanh"))
main <- glm(SUM~Var1+Var2+Var3, sum.data, family=poisson())
full <- glm(SUM~Var1*Var2*Var3, sum.data, family=poisson())
prediction(net.sum, list.glm=list(main=main, full=full))
```

Index

*Topic **neural**
compute, 3
confidence.interval, 4
gwplot, 6
neuralnet, 7
neuralnet-package, 2
plot.nn, 10
prediction, 12

compute, *3*, 3, *10*
confidence.interval, *3*, 4, *10*

gwplot, *3*, 6, *10*

neuralnet, *5*, *7*, 7, *11, 12*
neuralnet-package, 2

par, *6*, *11*
plot.default, *6*
plot.nn, *3*, *10*, 10
prediction, *3*, *10*, 12
print.nn (neuralnet), 7
