# Supplementary material for: Factors influencing SMEs CloudERP adoption: A test with generalized linear model and artificial neural network
Source: Data Brief. 2018 Jul 11;20:969–77. doi: 10.1016/j.dib.2018.07.012 (PMC6139002; doi:10.1016/j.dib.2018.07.012)
Supplement: Supplementary file 5 — Supplementary material [file mmc5.pdf]

Appendix

Computer efficacy (CE): I could complete the job using CloudERP
"if there was no one around to tell me what to do as I go"
"if I had never used a package like it before"
"if I had only the software manuals for reference"
"if I had seen someone else using it before trying it myself"
"if I could call someone for help if I got stuck"
"if someone else had helped me get started"
"if I had a lot of time to complete the job for which the software was provided"
"if I had just the built-in help facility for assistance"
"if someone showed me how to do it first"
"if I had used similar packages before this one to do the same job"

Organizational/Vendor support
"I know where to turn to when I need any assistance with our CloudERP system"
"In my company we get good technical support for our CloudERP system"
"We have extensive support to help with problems related to our CloudERP system"

Complexity (CMP)
"Using CloudERP system takes much time from my normal duties"
"Working with CloudERP is so complicated, it is difficult to understand what is going on"
"Using the CloudERP system involves much time doing mechanical operations (e.g., data input)"
"It takes too long to learn how to use CloudERP to make it worth the effort"

Compatibility (COM)
"Data captured in the CloudERP system and their format match my current data needs"
"The CloudERP system matches my current processing procedure"
"The changes caused by the adoption of CloudERP are compatible with the existing operating practices"
"The adoption of CloudERP is compatible with the firm's IT infrastructure"

Performance expectancy (PEY)
"I would find the CloudERP system useful in my job"
"Using the CloudERP system enables me to accomplish tasks more quickly"
"Using the CloudERP system increases my productivity"

Facilitating conditions (FC)
"I have the necessary resources to use CloudERP system"
"I have the necessary knowledge to use CloudERP system"
"If I have difficulty using CloudERP system, there will be professionals to help me"

Security (Information Integrity)
"Using CloudERP system would ensure the accuracy of the information handled"
"Using CloudERP systemwould ensure that the information is not deleted or modified"

Relative advantage (RA)
"CloudERP system would enable our enterprise to market our products/services in a better way"
"CloudERP system would enable our enterprise to communicate with our customers effectively"
"CloudERP system to reach our customers timeously with mobile marketing campaigns"
"CloudERP system would assist us to develop better relationships with our customers"

Perceived usefulness (PU)
"CloudERP system would enable me to accomplish tasks"
"CloudERP system would improve my job performance"
"CloudERP system would increase my productivity"
"CloudERP system would enhance my effectiveness"
"CloudERP system would make it easier to do my job"
"CloudERP system is useful in my job"

Perceive Ease of use (PE)
"Learning the CloudERP system is easy for me"
"Easy to get CloudERP system to do what I want to do"
"The CloudERP function is clear and understandable"
"CloudERP system is flexible to interact with"
"Easy to become skillful at using CloudERP system"
"CloudERP system is easy to use"

Intention to use (IU)/Adopt CloudERP
"I intend to use the CloudERP system for performing my job as often as needed"
"To the extent possible, I would frequently use the CloudERP system in my job"
